# Prevalence estimation of a rare disease with the French National Rare Disease Registry: example of TNF receptor associated periodic syndrome (TRAPS)

**DOI:** 10.1186/s13023-025-04086-4

**Published:** 2025-12-29

**Authors:** Adrien Subervie, Inès Elhani, Mathilde Labouret, Sophie Georgin-Lavialle, Eric Hachulla, Alexandre Belot, Arnaud Hot, Pierre Quartier, Achille Aouba, Alexandra Desdoits, David Saadoun, Marie-Elise Truchetet, Pascal Pillet, Guilaine Boursier, Ygal Benhamou, Martine Grall-Lerosey, Brigitte Granel, Olivier Fain, Viviane Queyrel, Alain Lescoat, Isabelle Melki, Veronique Hentgen

**Affiliations:** 1 French Reference Center for Autoinflammatory Diseases and Amyloïdosis, (CEREMAIA), Department of Pediatrics, Versailles Hospital, Versailles, France; 2 AP-HP, hôpital Tenon, Sorbonne université, Service de médecine interne, centre de référence des maladies auto-inflammatoires et des amyloses d’origine inflammatoire (CEREMAIA), Paris, 75020 France; 3 Université Paris-Cité, Paris, France; 4 Department of Internal Medicine and Clinical Immunology, Referral Centre for Rare Systemic Auto-Immune and Auto-Inflammatory Diseases (CeRAINOM), Univ. Lille, Inserm, CHU Lille, U1286 – INFINITE, University of Lille, Lille, F-59037 France; 5 Centre International de Recherche en Infectiologie, CIRI, Inserm, U1111, École Normale Supérieure de Lyon, Université Claude Bernard Lyon 1, Université de Lyon, Lyon, France; 6 Department of Clinical Immunology and Internal Medicine, University of Lyon 1, Hôpital Edouard Herriot, Hospices Civils de Lyon, Lyon, France; 7 Pediatric Immuno-Hematology and Rheumatology Unit, RAISE Rare Disease Reference Centre, Hopital Universitaire Necker-Enfants Malades, Assistance Publique-Hopitaux de Paris, Paris, France; 8 Department of Internal Medicine, Caen University Hospital, Caen, France; 9 Caen University-Normandie, Caen, France; 10 Department of Pediatric Surgery and Pediatric Rheumatology, CHU Caen Normandie, Caen, France; 11 Sorbonne Universités, Hôpitql Pitié Salpêtrière, Paris, France; 12 Département de médecine interne et d’immunologie clinique, Paris, France; 13 AP-HP, Centre de Référence des Maladies Auto-Immunes Systémiques Rares, French Reference Center for Autoinflammatory diseases and amyloidosis (CEREMAIA), Paris, F-75013 France; 14 INSERM, UMR 959, Paris, F-75013 France; 15 Bordeaux University Hospital, FHU ACRONIM, Place Amélie-Raba-Léon, Bordeaux, 33076 France; 16 CNRS UMR 5164 Immuno ConcEpT, Bordeaux University, Bât. 1B, 146 Rue Léo Saignat, Bordeaux, 33076 France; 17 Paediatric Department, Bordeaux University Hospital Pellegrin, Bordeaux, France; 18 Department of Molecular Genetics and Cytogenomics, Rare and Auto Inflammatory Diseases Unit, CHU Montpellier, University of Montpellier, Montpellier, France; 19 UNI Rouen U1096, Service de médecine interne, Normandie université, CHU Charles-Nicolle, Rouen, France; 20 F-CRIN INNOVTE Network, Saint-Etienne, France; 21 Department of Pediatrics, CHU Rouen, Rouen, France; 22 Internal Medicine Department, Hôpital Nord, Assistance Publique des Hôpitaux de Marseille (APHM), Aix-Marseille Université (AMU), Marseille, France; 23 Department of Internal Medicine, Inflammation-Immunopathology-Biotherapy Department (DHU i2B), Assistance Publique - Hôpitaux de Paris, Hôpital Saint-Antoine, 184, rue du Faubourg Saint-Antoine, Paris, 75012 France; 24 Department of Rheumatology, Nice University Hospital, Nice, France; 25 Department of Internal Medicine and Clinical Immunology, Rennes University Hospital, Rennes, France; 26 Université de Paris, Imagine Institute, Laboratory of Neurogenetics and Neuroinflammation, Paris, France; 27 Pediatric Hematology-Immunology and Rheumatology Department, Hôpital Necker-Enfants Malades, AP-HP Centre Université de Paris, Paris, France; 28 General Pediatrics-Infectious Diseases and Internal Medicine Department, Trousseau Hospital, AP-HP, Paris, Paris, France

**Keywords:** TRAPS, Rare diseases epidemiology, Rare diseases national registry, Rare diseases, BNDMR

## Abstract

**Background:**

Rare diseases (RD) have progressively emerged as public health priority in many countries. Epidemiological data are still lacking and the extraction of data from the public health system remains insufficient. In France, RD database set up in 2013 as Banque Nationale de Données de Maladies Rares (BNDMR). Patients’ information is provided by physician at each consultation and RD are classified according ORPHAcode. The status of each diagnosis can be entered as ‘confirmed’, ‘probable’ or ‘under investigation’, as well as related families.

**Objectives and methods:**

We aimed to test the reliability and quality of data for epidemiology by analyzing the data from a RD caused by autosomal dominant inheritance and with a univocal genetic diagnosis: TNF receptor-associated periodic syndrome (TRAPS). Patients were extracted on January 2023 and genetic files were retrieved from January to march 2023. All patients registered with a diagnosis of TRAPS were included.

**Results:**

We identified 132 patients who fulfilled inclusion criteria, among which 31 were excluded (missing data and duplicates). We analyzed 101 patients and their sequences of *TNFSRSF1A* gene. Pathogenic and likely pathogenic variants were found in 69% of patients, while the remaining 31% may rather represent undetermined systemic autoinflammatory disease. The main pathogenic variant found was T50M (c.236 G > T) while the main VUS was R92Q (c.362 G > A). For the patients entered as ‘confirmed’, only 44% of them had a pathogenic/likely pathogenic variant. We identified eight different families. We therefore estimated the minimum prevalence of TRAPS in France: 1/1 156 711.

**Conclusion:**

In the French National Rare Disease Registry, the quality of data remains a challenge, especially in monogenic diseases where the knowledge of the pathogenicity of variants and the number of gene involved is constantly increasing. Our study suggests that the data exported from the BNDMR needs important data correction to allow reliable epidemiologic studies in these diseases.

## Background

In numerous countries, rare diseases (RDs) have increasingly been identified as a significant global public health concern [1]. While the number of individuals diagnosed with a specific rare disease (RD) may be relatively low, the global population of persons living with a RD and in need of highly specialized healthcare is substantial [2, 3]. However, the epidemiology of RD still presents significant challenges. Diagnosis is often difficult, leading to misdiagnosis and inappropriate treatments. Training for the majority of health professionals is inadequate, and certain diseases are more prevalent in particular ethnic groups [4]. Furthermore, the definition of a RD varies across continents. In Europe, a RD is defined as having a prevalence of less than one in 2,000 inhabitants, whereas in North America, -according to the National Organization for Rare Disorders- a condition is considered rare if it affects fewer than 200,000 Americans. In light of these circumstances, it is indeed challenging to identify reliable data that can be used to assess the impact of rare diseases (RDs) on public health. The extraction of data from national public health systems for the estimation of the prevalence of RDs has already been demonstrated to be an inadequate approach [5–7]. In response to this challenge, several initiatives have been launched at the European and international level with the aim of establishing dedicated RDs national databases and high-quality registries based on the Orphanet classification of RDs [8–10].

The French National Rare Disease Registry (Banque nationale de données de maladies rares – BNDMR) is a national French registry established in 2013 [11]. The database collects a minimum data set, including diagnosis coded in accordance with the Orphanet nomenclature [8]. The objective of this repository is to provide France with a uniform collection of a minimal data set, which will document the care and health status of French patients with a RD and assess the impact of national health policy plans. A further objective of this database is to assess the prevalence of RD in the population. In France, all patients under the care of RD expert centres are required to be registered in the BNDMR. The database currently contains information on over one million patients [12] and 4,600 [13] different diseases. It is the responsibility of the attending physician to register patients and select the appropriate ORPHA code. It is reasonable to assume that the BNDMR will yield data of a high quality, given that the database is populated by medical experts and ORPHA codes are used.

In order to ensure that the data entered into a registry accurately reflects the epidemiological reality of a disease, it is essential that the registry includes a pathognomonic diagnostic test, whether clinical, biological or genetic. A significant number of RDs are devoid of a pathognomonic diagnostic test. Nevertheless, it is estimated that approximately 80% of RDs have a genetic origin. Consequently, genetic diagnosis may be the optimal approach for a number of these conditions, such as the rare hereditary autoinflammatory syndromes.

In autoinflammatory diseases (AID), as the understanding of molecular pathways related to genetic improves, genetic test become a major issue in their management. Even if the most AID have nonspecific clinical and biological features, their management, especially drug treatment, is however specific and needs an accurate diagnosis [14]. TNF-alpha receptor associated periodic syndrome (TRAPS) is an ultra-rare AID of autosomal dominant inheritance [15]. Although the symptoms are numerous and non-specific (long periodic fever, abdominal – chest – testicular pain, peri-orbital oedema, myalgia and rash), a definitive diagnosis can be made when a pathogenic mutation in the *TNFRSF1A* gene is identified [16, 17]. Although many variants have been described, only a few are considered pathogenic or likely pathogenic. The Public Infevers Database (https://infevers.umai-montpellier.fr/web/search.php?n=2) provides a comprehensive listing of all described variants and offers ongoing updates. The database classifies these variants according to their pathogenicity. This classification has been validated by the International Study Group for Systemic AIDs (INSAID) [18]. In 2019, Gattorno and al [19] proposed to admit the classification of TRAPS in patients with a variant in *TNFRSF1A* when associated with the following criteria: duration of episodes for at least > 7 days, myalgia, migratory rash, periorbital oedema and relatives affected.

Given the need of strong and reliable data about RDs, and some RDs’ complex diagnostic criteria, we aimed to appreciate BNDMR global reliabilty througth TRAPS data. A secondary objective was to estimate the prevalence of TRAPS in the French population.

## Methods

### The BNDMR

At each consultation, physicians from expert centres complete the patient’s data. Once a diagnosis has been either suspected or established, the physician enters the diagnosis into the BNDMR and selects the appropriate status: ‘under investigation’, ‘probable’, or ‘confirmed’. It is also possible to enter the family relation (and its degree) between different patients. It is the responsibility of the expert physician to update the data each time the patient encounters the French healthcare system.

### Legal considerations

In accordance with the procedures outlined in the BNDMR, a non-objection form was completed by the physician responsible for registering the patient and explanation about this non-interventional national study is given to the patient. Information regarding the data is available on the BNDMR website. In accordance with French legislation, each patient is entitled to request access and rectification of data and to object to the use of data for medical studies [20].

### Study population

The database was populated with all patients who had been diagnosed with TRAPS. The data were extracted on 23 January 2023. The BNDMR enables physicians to collate patients’ genetic data. However, as the data in question was absent from the majority of the files at the time of extraction, we proceeded to complete the genetic status for *TNFSFR1A* variants from January to March 2023 by requesting the precise genetic test results from the physician responsible for the file in question.

The first patients with TRAPS were entered at the year 2013 as the BNDMR was created.

The primary endpoint for evaluating the accuracy of the diagnosis rendered by the treating physician was the analysis of their variant of the *TNFRSF1A* gene, determined by Sanger or high-throughput frequency sequencing. *TNFRSF1A* variants were classified according to the Infevers database (https://infevers.umai-montpellier.fr) as pathogenic, likely pathogenic or of unknown significance [21].

To further refine the diagnosis entered in the database, we then considered the patient’s diagnostic status (i.e., under investigation, probable, or confirmed). Duplicate files were eliminated by means of a comparison of the patient identities associated with each expert centre. Finally, an evaluation of the sex ratio of patients in the database was conducted as a test of internal validity and to detect any possible declarative bias.

The prevalence has been calculated based on the number of inhabitants in France on 1 January 2023 [22], which was 68,246,000.

## Results

**Fig. 1:**
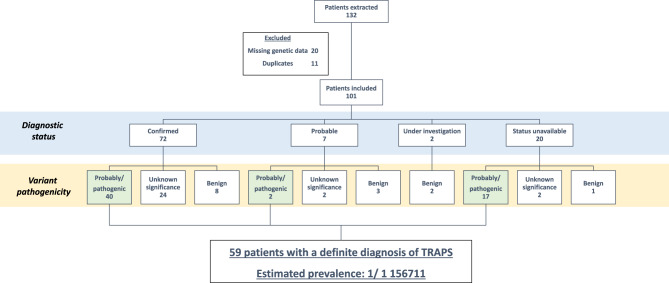
Flowchart of the study

At the time of extraction, a total of 132 patients with a diagnosis of TRAPS were registered in the database. The process of data extraction and cleaning was completed by four clinical research associates (CP, AK, ML, MH) over a period of 30 hours. Following a follow-up with the physicians, 20 patients were excluded due to incomplete genetic data, and an additional 11 patients were excluded due to the presence of duplicate entries (Fig. 1).

A total of 101 patients were included in the study, comprising 35 men and 66 women. *TNFRSF1A* was analysed using Sanger sequencing in 95 patients (94%) and high-throughput sequencing in 6 patients (6%). The sex ratio was 0.53.

For 20 patients, the clinician did not provide the diagnostic status, although 17 of them exhibit a pathogenic or likely pathogenic variant of *TNFRSF1A* gene.

### Variants of TNFRSF1A (Figure 2 and Table 1)

**Fig. 2:**
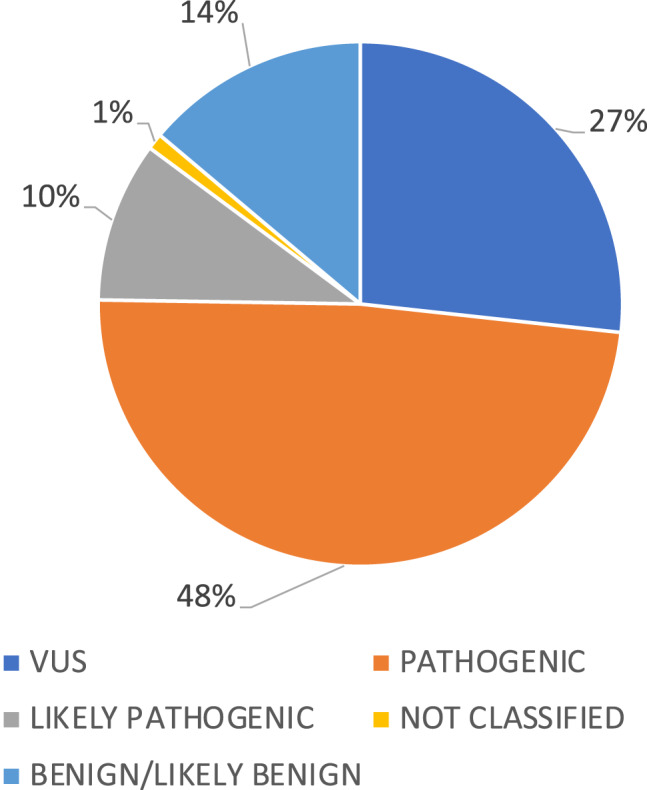
Pathogenicity classification of each variant of the TNFRSF1A gene in the study population

**Table 1:** Variants of *TNFRSF1A* gene in study population (excepted benign and likely benign variants)

Classification*	Number of patients	Variant	sequence
PATHOGENIC	24	T50M	c.236 G > T
	4	C30S	c.176 G > C
	3	C98R	c.379T > C
	2	C29S	c.173 G > C
	2	C30Y	c.176C > A
	2	C43Y	c.215 G > A
	2	C43S	c.215 G > C
	1	C30R	c.175T > C
	1	C30F	c.176 G > T
	1	C33Y	c.185 G > A
	1	C43F	c.215 G > T
	1	NA	c.241T > A
	1	C55R	c.250T > C
	1	C55S	c.251 G > C
	1	C70S	c.295T > A
	1	C70Y	c.296 G > A
LIKELY PATHOGENIC	4	L67P	c.287T > C
	3	Y106C	c.404A > G
	1	Y20C	c.146A > G
	1	D42E	c.213C > A
	1	H69fs	c.293_295del
	1	V125M	c.460 G > A
VARIANT OF UNKNOWN SIGNIFICANCE	22	R92Q	c.362 G > A
	2	P46L	c.224C > T
	1	D12E	c.123T > G
	1	D427E	c.1281C > A
	1	NA	p.503 G > T
UNCLASSIFIED	1	S116Del	c.347_349delCTT

*Pathogenicity as described in infevers database

The results of our study are presented in Table 1, which lists all the variants of the *TNFRSF1A* gene that were identified. A total of 59 patients (69%) exhibited either a pathogenic or a likely pathogenic variant in the *TNFRSF1A* gene, including 48 pathogenic and 11 likely pathogenic variants (56 and 13%, respectively). Twenty-seven patients (31%) exhibited variants of uncertain significance (VUS), with 22 displaying the R92Q (c.362 G > A) variant and 2 displaying the P46L (c.224C > T) variant. *TNFRSF1A* variants were not identified in 14 patients. An unclassified variant (c.347_349delCTT) was identified in a single patient.

Clinical data were available for 22 of the 26 (85%) patients with VUS, and only five of them (23%) fulfilled the EUROFEVER/PRINTO criteria.

### Diagnosis status and variant pathogenicity (Table 2 and Figure 2)

**Table 2:** Status of diagnosis and pathogenicity of *TNFRSF1A* gene variant

	Probable	Confirmed	Under Investigation	Total
VUS*	2	24	0	26
PATHOGENIC	1	32	0	33
LIKELY PATHOGENIC	1	8	0	9
UNCLASSIFIED	0	1	0	1
BENIGN/LIKELY BENIGN	3	7	2	12
Total	7	72	2	81

*VUS = variant of uncertain significance

The diagnosis status was registered for 81 patients (80%) and categorized as either ‘confirmed’, ‘probable’ or ‘under investigation’. Of these, 72 patients (89%) were confirmed to have the condition. Among the confirmed cases, only 40 patients displayed a pathogenic or likely pathogenic variant of *TNFRSF1A*, while 32 patients did not exhibit any known variant (44%).

### Family aggregation

We were able to identify twenty related patients, divided into eight different families. All patients within those families were entered and analyzed separately.

The families identified included between 2 and 3 patients. We found 6 males and 14 females among those families.

### Prevalence estimation

A minimal prevalence of TRAPS in France was estimated at 1/1,156,711, based on the 59 patients with a definite genetic diagnosis (pathogenic and likely pathogenic variants), such as 2021 EULAR/ACR recommendation [16]. If we included patient with VUS of *TNFRSF1A*, the estimate prevalence is 1/793,558.

## Discussion

### Main result

Despite the BNDMR’s reliance on expert physicians and adherence to international coding standards for RDs [8, 11], our findings indicate that the BNDMR does not provide a direct estimation of TRAPS prevalence. Indeed, a round of callbacks was necessary to obtain the requisite data to calculate the epidemiological indicators. Furthermore, only 69% of the documented patients satisfied the recommended genetic criteria [16] for a definitive diagnosis, while 31% exhibited a variant of uncertain significance in *TNFRSF1A*. The accuracy of diagnoses was not superior in patients with a “confirmed” diagnosis in the database. This observation could be explained by a number of factors.

Firstly, even within a network dedicated to RD research, there may be a lack of awareness regarding this ultra-rare disease and the most recent classification of pathogenicity of variants. It is thus possible that *TNFRSF1A* variants have been consistently interpreted as pathogenic by the treating physician. In the context of TRAPS, the potential relevance of the R92Q variant is heightened by the ongoing controversy surrounding its pathogenicity. Despite the initial characterization of the variant as the underlying cause of the TRAPS phenotype [23], subsequent research has revealed that R92Q is prevalent in the general population and does not consistently manifest in family members. Consequently, R92Q is currently listed as a VUS in the Infevers database, despite the fact that some researchers still regard it as a low-penetrance variant [23]. The aforementioned controversy, in conjunction with the evolving perception of this variant, may have contributed to the high prevalence of VUS observed in patients with a “confirmed” TRAPS diagnosis in the BNDMR. A similar argument can be made with regard to other downgraded variants of the *TNFRSF1A* gene.

Moreover, between approximately 1999 and 2019, the diagnosis of TRAPS was based on Sanger sequencing of known hotspot variants, and the genetic forms described were exclusively germline mutations. In the last five years, new-generation sequencing techniques have been developed to identify variants in other exons and to detect somatic forms. It is now recognised that a diagnosis of TRAPS necessitates the presence of a genetic mutation, at least in the somatic state [16]. Prior to the advent of next-generation sequencing (NGS) techniques, a purely clinical diagnosis was possible. However, it is possible that these diagnostic changes were not considered by physicians, as evidenced by the high percentage of exclusive Sanger sequencing in patients without identified genetic mutations.

A second potential reason may be associated with the primary objective of the BNDMR. It can be stated that the database is primarily used for the purpose of documenting the impact of RD on the French healthcare system, and is furthermore employed as a means of funding expert centres. It is therefore recommended that physicians enter patients into the database at each contact with an expert centre, even if the diagnosis of a RD is not yet certain. It is possible that suspected TRAPS diagnoses may be recorded in the database at the initial point of contact. However, given that the results of genetic testing are not available until several months later, it is possible that physicians may not update the diagnoses initially entered. Currently, BNDMR is only a database for RDs diagnosis, with minimal clinical features (not mandatory at each record) and no biobank integrated. To address this issue, an automated link between the BNDMR and the two national whole genome sequencing platforms databases (Seqoia and Auragen) [24, 25] is planned. It is anticipated that this update will enhance the quality of genetic BNDMR data, contingent on the implementation of the revised diagnosis.

The TRAPS model proved an effective means of evaluating the quality of data pertaining to BNDMR-reported diagnoses, given that its genetic diagnosis is unambiguous [16]. The assessment of the accuracy of recorded data in other RDs will be a far more challenging undertaking, given that diagnosis is based on a range of clinical and biological features. Additionally, it represents one of the four historical monogenic AIDs, initially described at the end of the 1990s, for which genetic knowledge has significantly advanced in recent years. Furthermore, the accuracy of the definitive diagnosis has evolved, and patients may have been misdiagnosed with TRAPS prior to the advent of new genetic insights into the disease.

A number of potential avenues for enhancing the diagnostic precision of patients included in the BNDMR database have been identified. One such avenue is the introduction of a requirement for physicians to complete the diagnosis criteria or the outcome of a multidisciplinary consultation meeting when entering a patient into the database. Furthermore, the software could be programmed to prompt physicians to re-evaluate the diagnosis entered. Ultimately, the database must be modular and rely on subsequent studies to correct for changes in variant pathogenicity classification.

The first solution may result in underreporting due to the limited time available for research by practitioners entering data into the database. The second solution could potentially limit the scope for studies on RDs via the BNDMR. Both strategies require the allocation of dedicated time and personnel, as well as training in data implementation. One potential solution to these challenges is to incorporate links to existing specialized RDs databases, such as the JIRcohort for AIDs, into the BNDMR. These databases often contain more detailed and longitudinal patient data, which could enhance the accuracy and completeness of the BNDMR [26, 27].

As we discussed, the exhaustivity of BNDMR is not certain for multiples reasons. This might explain the high female to male sex ratio in the whole population of the study, as well for the related members in the eight identified families. The de novo variant appearance in *TNFRSF1A* gene is still not well known.

We elected to calculate the estimated prevalence of TRAPS in France, basing our calculations on patients who displayed likely pathogenic or pathogenic variants in *TNFRSF1A*, in accordance with the European recommendations [16]. We could not be sure of including all patients who met the EUROFEVER/PRINTO criteria because of the limitations of the BNDMR in terms of clinical features, and we may even have underestimated the true prevalence of TRAPS in France. Our findings suggest that TRAPS affects at least 1 in 1,150,000 individuals in the French population (1 in 800,000 VUS included).

In 2009, Lainka et al. [28] conducted a study on the epidemiology of TRAPS in German children (aged < 16 years). Their findings indicated a prevalence of 8.96 per 10^6^ children, which is 11 times more frequent than our estimation (7 times more with VUS included). Although the populations are comparable (predominantly European in origin), this study considers only paediatric patients, whereas our study focuses on the general population. As a consequence of the methodology employed (a monthly survey to pediatricians and rheumatologists, and genetic laboratories), underreporting represents a significant challenge, as it does for our study (data provided in the BNDMR solely by RD reference centres). However, 83% of patients in their cohort were found to harbour the frequent R92Q variant, which was excluded from our prevalence analysis due to its uncertain significance. It can be reasonably assumed that the discrepancy between the two studies is due to the differing methodologies employed.

### Strength and limitations

We studied about a very rare disease. Given that main of patients with TRAPS had at least one visit in expert centers, which all enter data in BNDMR, we can assume that the completeness of the data is as high as possible.

However, no patient has genetic diagnosis fulfilled at extraction and we had to retrieved genetic data from clinical files at each center. This is a major bias in this study and reflects the weakness of the raw data from the BNDMR.

In addition, it is possible that the data in the database were not entered by the expert physician. Indeed, in some centres, data is entered by other professionals, such as residents, or clinical research assistants. This could explain the presence of incorrect diagnosis data. Further analysis could investigate the impact of the status of the individual entering the data on the accuracy of diagnoses.

Finally, diagnosis of TRAPS is not easy in daily practice. Indeed, we chose to stick to the EULAR/ACR criteria while number of physician used EUROFEVER/PRINTO criteria from 2013 to 2022. This might be a reason why many patients were diagnosed with TRAPS and had VUS or non-pathogenic variant of *TNFRSF1A*

## Conclusions

Our study shows that the use of data from the French National Register of Rare Diseases (BNDMR) for clinical research necessitates a return to the source of medical records to guarantee the reliability of epidemiological data, particularly when the analysis pertains to a genetic disease associated with a gene that has numerous variants of unproven pathogenicity. However, our results cannot be extended to all RDs, given the unspecific clinical and biological features of TRAPS and the most of AIDs. Nevertheless, the database is proving to be an effective tool for identifying centres where patients with rare/ultra-rare diseases are managed and could be contacted for translational studies, epidemiological research or clinical trials.

## Data Availability

The datasets used and/or analysed during the current study are available from the corresponding author on reasonable request.

## Abbreviations

BNDMR: Banque nationale de données de maladies rares: French national rare diseases database
RD: Rare diseases
VUS: Variant of uncertain significance
TRAPS: TNF-Receptor Associated Periodic Syndrome
